# Regulating programmed cell death in plant cells: Intracellular acidification plays a pivotal role together with calcium signaling

**DOI:** 10.1093/plcell/koae245

**Published:** 2024-08-28

**Authors:** Maurice Bosch, Vernonica Franklin-Tong

**Affiliations:** Institute of Biological, Environmental and Rural Sciences (IBERS), Aberystwyth University, Plas Gogerddan, Aberystwyth SY23 3EE, UK; School of Biosciences, College of Life and Environmental Sciences, University of Birmingham, Edgbaston, Birmingham B15 2TT, UK

## Abstract

Programmed cell death (PCD) occurs in different tissues in response to a number of different signals in plant cells. Drawing from work in several different contexts, including root-cap cell differentiation, plant response to biotic and abiotic stress, and some self-incompatibility (SI) systems, the data suggest that, despite differences, there are underlying commonalities in the early decision-making stages of PCD. Here, we focus on how 2 cellular events, increased [Ca^2+^]_cyt_ levels and cytosolic acidification, appear to act as early signals involved in regulating both developmental and stimulus-induced PCD in plant cells.

## Introduction

Programmed cell death (PCD) is an active process that selectively eliminates unwanted cells. In plants, PCD can be triggered either as part of a developmental program or induced by a range of stimuli. Developmental PCD (dPCD) occurs naturally during specific growth stages. Examples include tracheary element differentiation, the development of endosperm and aleurone cells in cereals, tapetum PCD during pollen development, trichome formation, leaf morphogenesis and senescence, and terminal differentiation of the root cap (Zhang and Yang 2014). Stimulus-induced PCD (sPCD), conversely, is activated by external stimuli. This can involve interactions between different cells through receptor–ligand mechanisms to specify a recognition event, resulting in either acceptance or rejection, with rejection resulting in PCD of specific cells. Examples of sPCD include host–pathogen interactions triggered by an innate immunity system, resulting in a hypersensitive response (HR), and some self-incompatibility (SI) systems, which reject incompatible pollen. External abiotic stresses, such as drought, heat, UV light, high salinity/osmotic stress, and toxicity from heavy metals, can also trigger sPCD (Petrov et al. 2015; Zhang et al. 2022).

It is thought that there are several types of PCDs in plants, and various attempts have been made to categorize them; see, for example, Mur et al. (2007), Reape et al. (2008), van Doorn et al. (2011), and Kacprzyk et al. (2024). One was based on the different morphological characteristics of PCD (van Doorn et al. 2011) and includes what has been termed as “vacuolar PCD” (vPCD). Execution of vPCD (sometimes called “autolytic” PCD) involves the rupture or collapse of the vacuolar membrane (tonoplast), which results in a sudden release of vacuolar hydrolases into the cytosol. The collapse of the vacuole is necessary for the breakdown of organelles and finally the plasma membrane (PM; van Doorn et al. 2011). A classic example of vPCD is the PCD of tracheary elements as part of an overall xylem maturation program (see Bollhöner et al. 2012). PCD in tracheary elements exhibits progressive degeneration of the nucleus, vacuole, plastids, mitochondria, and endoplasmic reticulum (ER) and, finally, the removal of the PM (Fukuda 1996). Another form of vPCD is observed in some forms of the HR, sometimes referred to as “nonautolytic” vPCD, where the vacuolar membrane remains intact for some time and there is no rapid clearance of the cytoplasm (Hatsugai et al. 2009). In contrast, necrosis is a rapid form of PCD, characterized by mitochondrial dysfunction, early rupture of the PM, and consequential cellular disassembly with loss of intracellular content (van Doorn et al. 2011). However, many types of plant PCD do not fall easily into a specific category based on morphological features and were classified under the awkward category of “mixed and atypical” (van Doorn et al. 2011); these include dPCD in starchy cereal endosperm and in the root cap during differentiation, as well as sPCD involving receptor–ligand-type interactions, such as the HR and SI in *Papaver* (see van Doorn et al. 2011). Categorizing the various types of PCD is challenging and sometimes confusing, as some exhibit shared features but differ in other aspects (see Kacprzyk et al. 2024).

In the last couple of decades, considerable progress has been made in our understanding of the signals and cellular responses triggered during plant PCD in different systems. This can involve increases in cytosolic-free Ca^2+^ ([Ca^2+^]_cyt_), reactive oxygen species (ROS), and MAP kinases (MAPKs), which act in a network to activate a PCD signaling cascade (Lecourieux et al. 2006; Peng et al. 2018), leading to changes in organelle structure and permeability, and ultimately resulting in cell death. Numerous examples of PCD signaling have been documented; however, despite significant advances, it remains unclear whether common core cellular mechanisms participate in regulating different types of PCD in plants.

**Figure 1.**
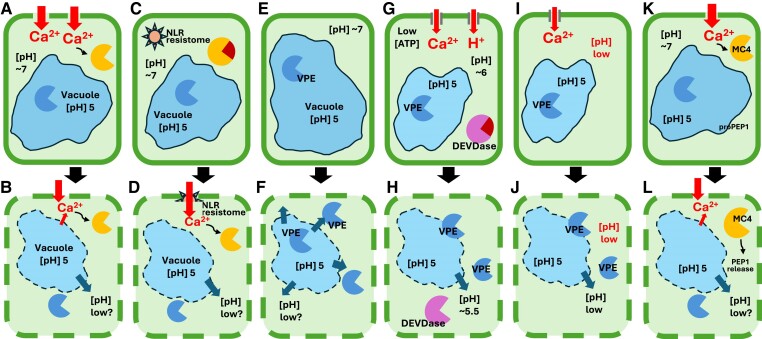
Examples of rapid and delayed vacuolar breakdown and cytosolic acidification leading to cellular dismantling during PCD. **A, B)** PTI triggers rapid Ca^2+^ influx during the immune response. The involvement of several different Ca^2+^ channels mediating Ca^2+^ influx have been identified (Xu et al. 2022). Increased [Ca^2+^]_cyt_ is thought to activate Ca^2+^-sensitive metacaspases present in the cytosol **(A)** and this leads to cellular dismantling due to proteolysis **(B)**, including subsequent loss of vacuolar integrity. Cytosolic acidification has not been measured, but it is assumed that the cytosol becomes acidic after vacuole integrity is lost. **C, D)** Effector-triggered immunity triggers rapid signaling responses, including Ca^2+^ influx. The activated NLR resistome translocates to the PM where it functions as a noncanonical Ca^2+^ channel, resulting in Ca^2+^ influx and PCD involving the HR (Xu et al. 2022). In an uninduced cell **(C)**, the resistome is cytosolic. After pathogen perception **(D)**, the NLR resistome is inserted into the PM, where it acts as a Ca^2+^ channel allowing Ca^2+^ influx. This is thought to activate Ca^2+^-sensitive metacaspases present in the cytosol, and cellular dismantling occurs due to proteolysis, including loss of vacuolar integrity. Cytosolic acidification has not been measured, but it is assumed that the cytosol becomes acidic after vacuole integrity is lost. **E, F)** Rapid vacuolar breakdown. Intact cell **(E)**. The intracellular pH of plant cells is strictly controlled. Under normal cellular conditions, cytosolic pH ([pH]_cyt_) is typically ∼7 and vacuolar pH is ∼5.5. The cytosol contains many inactive proteases; the intact vacuole contains active VPE (YVADase). Cytosolic-free Ca^2+^ ([Ca^2+^]_cyt_) is low. Rapid vacuolar breakdown involves classic “autolytic” vPCD **(F)**. The vacuolar membrane (tonoplast) ruptures/collapses, suddenly releasing vacuolar hydrolases, e.g. VPE, into the cytosol. This causes rapid dismantling of the cytosolic contents through hydrolysis. Cytosolic pH will acidify due to leakage from the vacuole; this is assumed but has not been measured. An example of this is dPCD in tracheary elements as a part of an overall xylem maturation program; see Fukuda (1996) and Bollhöner et al. (2012). **G, H)** Delayed vacuolar breakdown. Rapid acidification prior to loss of membrane integrity in the incompatible Papaver pollen SI response. Rapid influx of Ca^2+^ and H^+^ are triggered **(G)** and the cytosolic pH rapidly acidifies; see Franklin-Tong et al. (1997), Wilkins et al. (2015), and Wang et al. (2022b). After SI induction, the [pH]_cyt_ reaches ∼pH 6 within 10 min, but membrane integrity is retained at this stage. DEVDase in the cytosol is inactive; it is assumed that the vacuolar VPE (YVADase) is active in the acidic vacuolar compartment. Several hours later **(H)**, the DEVDase, which requires an optimal pH of 5, is activated, with peak activity at 5 h. Loss of vacuolar integrity (which, it is assumed, releases active VPE) must occur several hours after the initial cytosolic acidification (Bosch and Franklin-Tong 2007). **I, J)** Delayed vacuolar breakdown. Rapid acidification prior to loss of membrane integrity in the LRC system in roots; see Fendrych et al. (2014) and Wang et al. (2024). Rapid influx of Ca^2+^ is triggered **(I)** and the cytosolic pH rapidly acidifies, although how this is achieved is not known. At this stage, PM integrity is retained. Soon after increases in Ca^2+^ and H^+^, organelles begin to break down (not shown), starting with the mitochondria, followed soon after by collapse of the nucleus and ER. Finally, the large central vacuole loses integrity **(J)**, and the PM becomes permeable. It is assumed that the loss of vacuolar integrity will result in the release of active VPE into the cytosol. **K, L)** Artificial damage to cells triggers Ca^2+^ influx, activating Ca^2+^-dependent metacaspases. Early response to cellular damage **(K)**. Loss of PM integrity results in high and prolonged influx of extracellular Ca^2+^ into the cytosol, sufficient to activate Ca^2+^-dependent metacaspases (which do not have an acidic pH optimum for activity) in the cytosol. After loss of PM integrity **(L)**, activation of MC4 is triggered by prolonged high levels of [Ca^2+^]_cyt_; this causes cleavage of ProPep1 to Pep1, which is released from the vacuolar membrane; see Hander et al. (2019). Cellular dismantling occurs due to proteolysis, including loss of vacuolar integrity. Cytosolic acidification has not been measured.

[Ca^2+^]_cyt_ has been recognized as a pivotal second messenger in plant signaling for several decades. Its involvement has been described for a plethora of plant responses, both biotic and abiotic, including the PCD response (Sanders et al. 1999; Lecourieux et al. 2002, 2006; Brownlee and Hetherington 2011; Köster et al. 2022; Wang et al. 2024). We now have a relatively good understanding of the key components involved in regulating alterations in [Ca^2+^]_cyt_ in plants; see, for example, Sze et al. (2000), Dodd et al. (2010), Chen et al. (2015), Edel et al. (2017), Kudla et al. (2018), Tian et al. (2020), and Luan and Wang (2021) for reviews. Alterations in [Ca^2+^]_cyt_ have long been established as one of the first triggers that play a pivotal role in PCD. For example, during basal defense and the HR, increases in [Ca^2+^]_cyt_ are triggered soon after pathogen perception (Lecourieux et al. 2006; Ma and Berkowitz 2007); see Ren et al. (2021) for a recent review. Indeed, it has been proposed that Ca^2+^ signatures may be decoded by metacaspases to transduce Ca^2+^ signals to activate distinct PCD response pathways (Zhu et al. 2020). In recent years, considerable progress has been made in understanding the role of diverse calcium channels in plant immunity and cell survival. Pattern-triggered immunity (PTI) utilizes a range of different Ca^2+^ channels to mediate Ca^2+^ influx during the immune response (Fig. 1, A and B). These include cyclic nucleotide-gated ion channels, glutamate receptor-like proteins, reduced hyperosmolality-induced [Ca^2+^]_cyt_ increase channels, 2-pore channels, and annexins (Xu et al. 2022). A major recent breakthrough has revealed that a class of plant immune receptors that trigger an HR, the ZAR1 nucleotide-binding leucine-rich repeat receptor (NLR) resistome, function as noncanonical Ca^2+^-permeable channels (Fig. 1, C and D). This reveals new perspectives on how plant immune receptors trigger PCD through Ca^2+^ signaling (Bi et al. 2021; Xu et al. 2022) and suggests that a major conserved feature of these responses involves Ca^2+^ influx, even though the channels themselves are not conserved (Fig. 1, A to D). Thus, it is clear that a critical step involved in triggering many types of PCD is Ca^2+^ influx.

The intracellular pH of plant cells is strictly controlled. Under normal cellular conditions, cytosolic pH ([pH]_cyt_) is typically alkaline between ∼6.9 and 7.5 pH units (Kurkdjian and Guern 1989; Felle 2001; Ishizawa 2014). In contrast, the vacuole and apoplast are highly acidic, around pH 5.5 (Katsuhara et al. 1989; Fig. 1E). The majority of measurements of pH during normal growth and development in plant cells report relatively modest, transient changes in [pH]_cyt_ of ∼0.4 to 0.7 pH units; for example, during gravitropic responses, root hair growth, and pollen tube elongation. Studies investigating [pH]_cyt_ in response to physiologically relevant signals, including decreases in light intensity, and addition of elicitors, hormones, and other treatments, also report small transient alterations in a similar range; see Wilkins et al. (2015) and Behera et al. (2018). These observations have led to the proposal that [pH]_cyt_ acts as a second messenger (Behera et al. 2018). Intracellular pH regulation involves complex interactions of ion transport, including H^+^-buffering, H^+^-consuming, and H^+^-producing reactions (Felle 2001). The control of transmembrane pH gradients requires a delicate interplay between H^+^ fluxes and energy metabolism (Wegner and Shabala 2020). In recent years, an interest in intracellular pH and the idea that it plays a signaling role has been revived (Raghavendra et al. 2023). The activity of PM H^+^-ATPases is critical for the homeostasis of cellular pH (Falhof et al. 2016; Kinoshita and Kinoshita 2022). Apoplastic pH, which is usually acidic under normal growth conditions, becomes alkaline during abiotic/biotic stress and is thought to be crucial in plant responses to hormones and other signals (Gámez-Arjona et al. 2022). Modulating apoplastic pH can cause changes in cell wall components, ion uptake, Ca^2+^, and ROS (Gámez-Arjona et al. 2022). It is becoming increasingly apparent that there is a fundamental link between Ca^2+^ and pH dynamics in plant cells, although it remains unclear whether pH changes are the cause or the consequence of the dynamic interplay between Ca^2+^ and H^+^ (Behera et al. 2018; Stéger and Palmgren 2022).

Although links between Ca^2+^ and H^+^ transport and the idea that they both act as second messengers are not new [see, for example, Hermann and Felle (1995) and Felle (2001)], with advances in imaging technology, the importance of protons and intracellular acidification to integrate a variety of signaling networks is becoming increasingly appreciated (Hermann and Felle 1995; Roos et al. 1998; Monshausen et al. 2011; Michard et al. 2017; Behera et al. 2018). Intracellular calcium and pH signaling are now understood to be tightly intertwined. For example, low pH has been shown to shape the Ca^2+^ signature of microbe-associated molecular patterns, such as flg22 (Westphal et al. 2019). Hence, where Ca^2+^ fluxes are observed, the effects of pH should also be considered. It has recently been proposed that Ca^2+^ signatures act in concert with pH signatures, possibly providing an additional layer of cellular signal transduction to tailor signal specificity (Behera et al. 2018). Another pivotal class of signaling molecules involved in regulating PCD is ROS. Respiratory burst oxidase homologs (RBOHs), which are plant-specific NADPH oxidases, have been identified as pivotal components, integrating calcium signaling and protein phosphorylation with ROS production in many biological processes including PCD (Van Breusegem and Dat 2006; Suzuki et al. 2011; Marino et al. 2012; Xie et al. 2014; Petrov et al. 2015; Yu et al. 2017). It is also becoming apparent that there is significant interplay between Ca^2+^ and ROS (Marcec et al. 2019). Although both play a critical role in signaling to PCD, the exact mechanisms by which they interact and coordinate these processes remain to be established; see Dangl et al. (1996) and Stael et al. (2015).

Here, we review studies of cytosolic acidification during PCD in various plant systems, suggesting that there may be common signaling mechanisms that regulate plant cell death during both sPCD and dPCD. We mostly focus on the evidence for Ca^2+^ signaling and acidification and how they may operate to regulate PCD, rather than later consequences. Taken together, the data suggest that cellular events involving cytosolic acidification, which occurs earlier and independently of vacuolar breakdown, trigger PCD in several plant systems.

## Cytosolic acidification: A PCD trigger often assumed, but rarely measured

Vacuolar rupture has long been known to be a key feature in many types of PCDs in plants. For instance, in the classic textbook example of dPCD in Zinnia, where xylem is formed from tracheary elements, the vacuole is known to suddenly rupture (Groover et al. 1997; Obara et al. 2001); see Bollhöner et al. (2012) for a review. This would almost certainly cause the [pH]_cyt_ to drop rapidly (Fig. 1F), as vacuoles are highly acidic; however, in most cases, this phenomenon has been proposed or assumed (Groover et al. 1997; Obara et al. 2001) but not measured.

One of the earliest studies to measure cytosolic acidification was in connection with SI in *Papaver rhoeas*. SI in this system is triggered by the interaction of a small cysteine-rich ligand (PrsS), secreted by the female pistil tissue, with a small “receptor-like” transmembrane protein (PrpS) carried by the pollen. Cognate interaction of these *S*-determinants triggers an incompatible response that ultimately results in PCD (Thomas and Franklin-Tong 2004). These studies used the ratiometric pH indicator 2,7-bis-(2-carboxyethyl)-5-(and-6)-carboxyfluorescein acetoxymethyl ester to measure cytosolic pH in incompatible pollen tubes. After SI-induced PCD, the [pH]_cyt_ of pollen tubes was measured at pH 5.5 (Bosch and Franklin-Tong 2007). Subsequent measurements of the temporal profile of these alterations revealed a surprisingly rapid reduction in [pH]_cyt_, reaching pH 6.4 within 10 min of SI induction and stabilizing at pH 5.5 by 60 min (Wilkins et al. 2015). The first detectable events triggered by the *Papaver* SI process were increases in [Ca^2+^]_cyt_ (Franklin-Tong et al. 1993, 1995) involving Ca^2+^ influx (Franklin-Tong et al. 2002), followed soon after by cytosolic acidification involving a rapid influx of H^+^ ions (Wilkins et al. 2015; Wang et al. 2022b; Fig. 1, G and H).

In another study, it was shown that the fluorescence of plant cells expressing yellow fluorescent protein (YFP) was greatly reduced during PCD (Young et al. 2010). Artificially inducing PCD by expressing the mammalian proapoptotic gene *BAX* in onion epidermal cells triggered several key PCD features, including loss of mitochondrial membrane potential and VADase caspase-like activity. Using loss of YFP fluorescence to monitor cytosolic acidification, the authors measured a drop in [pH]_cyt_ triggered by *BAX*-induced PCD in these cells; [pH]_cyt_ dropped from 7.3 to pH 5.7.

Using a pH-sensitive GFP variant, a large drop in [pH]_cyt_ was observed during the execution of dPCD in the lateral root cap (LRC) of *Arabidopsis thaliana* (Fendrych et al. 2014; Wang et al. 2024). Although the exact magnitude of the pH change was not calibrated, cytosolic acidification occurred prior to vacuolar collapse (Fendrych et al. 2014; Wang et al. 2024; Fig. 1, I and J). They also showed that preventing intracellular acidification by buffering reduced the frequency of PCD, while artificially reducing [pH]_cyt_ to pH 5.8 in these LRC cells increased the frequency of cell deaths (Fendrych et al. 2014). It was subsequently shown that in the LRC system, elevated intracellular Ca^2+^ and H^+^ levels are sufficient to trigger cell death execution in terminally differentiated root-cap cells (Wang et al. 2024). The authors proposed that these ion fluxes act as PCD-triggering signals (see also Chakraborty 2023).

Together, these studies provide strong evidence that cytosolic acidification may play a pivotal role as a signal or mechanism pushing the cell into PCD. They also raise the idea that there is interplay between [pH]_cyt_ and [Ca^2+^]_cyt_ signaling in regulating this process.

## A functional role for cytosolic acidification in regulating PCD?

Cytosolic acidification is clearly a key feature of plant PCD, prompting the question of its functional role in this process. A key role for this type of large cytosolic acidification is likely to be the activation of proteases involved in pushing the cell into the final stages of PCD execution. Many plant caspase-like enzymes have an acidic pH optimum, exhibiting no activity at the normal physiological [pH]_cyt_ of ∼pH 7. For example, vacuolar processing enzyme (VPE), a cysteine protease with caspase-1-like activity located in the vacuole, exhibits maximal activity at acidic pH. It is well established that some types of vPCD utilize VPE, which plays a pivotal role in triggering vacuolar membrane rupture/collapse leading to PCD in the immune response (Hatsugai et al. 2004, 2006, 2015; Wleklik and Borek 2023; Fig. 1F). VPE-mediated disruption of the vacuolar membranes, releasing the vacuolar contents, including hydrolytic enzymes, into the cytoplasm, leads to PCD, while *VPE*-silenced plants did not undergo vacuolar membrane disintegration or cell death (Hatsugai et al. 2004). Thus, VPE is responsible for triggering the degradation of the intracellular contents (van Doorn et al. 2011; Hatsugai et al. 2015), resulting in cell death (Fig. 1F). VPEs are known to be involved in various types of PCDs, including developmental processes, senescence, HR, and hormone signaling (Hatsugai et al. 2015). However, exactly how VPEs control vacuolar rupture remains unclear (Hatsugai et al. 2015). Nevertheless, this VPE-dependent vacuolar leakiness is upstream of, and required for, subsequent DEVDase/caspase-3-like activation in some types of PCD (see Li et al. 2012).

Cytosolic metacaspases play key roles in mediating PCD. Their pH optima vary significantly. For example, some metacaspases have an acidic pH optimum (e.g. pH 5.0 to 5.5 for AtMC9) and have no activity at normal physiological cytosolic pH (Vercammen et al. 2004). This requirement for acidic pH suggests that cytosolic acidification activates metacaspases to achieve cell death in certain plant PCD systems. However, many metacaspases do not require acidic conditions. For example, AtMC4 has an optimal pH of 7.5 to 8.0 (Vercammen et al. 2004). Like most metacaspases, AtMC4 requires high levels of Ca^2+^ for its activation, implicating Ca^2+^ as a pivotal upstream signal (Watanabe and Lam 2011; Hander et al. 2019). Thus, although AtMC4 is constitutively expressed in the cytosol and does not require an acidic pH, it remains inactive at physiological levels of [Ca^2+^]_cyt_ and is only activated by large, sustained increases in [Ca^2+^]_cyt_. The activation of AtMC4 after prolonged high levels of [Ca^2+^]_cyt_ due to Ca^2+^ influx induced the release of a peptide, Pep1, from the vacuolar membrane, which can then help initiate an immune-like response (Hander et al. 2019; Fig. 1, K and L). The recent determination of AtMC4's crystal structure has provided insights into how Ca^2+^ triggers its activation (Zhu et al. 2020). Similarly, the metacaspase mcII-Pa, which is required for induction of autophagy in a vPCD death pathway (Minina et al. 2013, 2014), is also active at pH 7.0 (Bozhkov et al. 2005) and requires Ca^2+^ for its activation. Therefore, cytosolic acidification is not likely to play a major role in PCD involving these metacaspases (Fig. 1, A to D).

Although the identities of many plant caspase-like enzymes remain unknown, several activities have been identified. In onion epidermal cells expressing BAX, a drop in [pH]_cyt_ was shown to correlate with induced VADase caspase-like activity (Young et al. 2010). This implicates a pivotal role for cytosolic acidification in the activation of caspase-like activities during PCD in this system. In the *Papaver* SI system, PCD involves a DEVDase/caspase-3-like activity (Thomas and Franklin-Tong 2004; Bosch and Franklin-Tong 2007). Several caspase-like activities (DEVDase, VEIDase, and LEVDase) were stimulated by SI in incompatible *Papaver* pollen tubes; the optimal pH for these SI-induced caspase-like activities is very narrow, with peak activity at pH 5 (Bosch and Franklin-Tong 2007). Hence, these enzymes are inactive in the normally growing pollen tubes, which have a [pH]_cyt_ of ∼pH 6.8, and require cytosolic acidification to become activated. Notably, the cytosolic acidification observed during SI fits the pH optima for the SI-induced caspase activities very closely. Moreover, by manipulating the [pH]_cyt_ of the pollen tubes in vivo, it was demonstrated that [pH]_cyt_ acidification is essential for DEVDase activation and SI-induced PCD (Wilkins et al. 2015). Another DEVDase, *A. thaliana* cathepsin B3 (AtCathB3), which is implicated in several abiotic stresses in Arabidopsis seedlings and protoplasts, including ultraviolet C, oxidative stress, and ER stress, also has optimal activity at pH 5.5 (Ge et al. 2016). This suggests that this acidic requirement for optimal activity of plant DEVDase enzymes may be a general phenomenon. However, a VEIDase activity involved in dPCD required for embryo pattern formation in Norway spruce (*Picea abies*) exhibits 2 optimal activities at ∼pH 7 and pH 4 (Bozhkov et al. 2004). It is not yet known whether the dPCD of the LRC system requires caspase-like activities or what their identities might be; this will be an interesting area to investigate in the future.

MAPKs regulate a variety of cellular processes in eukaryotes, including PCD. It has been shown that the toxin 2,4-D-triggered cytosolic acidification in tobacco cells, decreasing by >1.5 pH units within 15 min, which triggered MAPK activation (Tena and Renaudin 1998). Artificial acidification using butyric acid also activated MAPK. The authors estimated that MAPK activation was achieved when the [pH]_cyt_ was decreased by >0.4 pH units. This was likely the first evidence suggesting that [pH]_cyt_ may act as a second messenger, signaling to MAPK cascades. However, to our knowledge, how this cytosolic acidification is achieved and how it activates MAPKs remains unknown. Interestingly, it has recently been shown that [pH]_cyt_ controls MAPK signaling and pathogenicity in the fungal pathogen *Fusarium oxysporum* (Fernandes et al. 2023), suggesting that these mechanisms may be widely utilized in eukaryotic cells.

Cellular activity is regulated at many levels and by many components. The functional properties of most proteins, including their activity, stability, associations, and subcellular translocation, are greatly affected by pH (Talley and Alexov 2010). It is therefore likely that cytosolic acidification results in a general stress response, which contributes to pushing the cell into PCD. For example, in the *Papaver* SI system, a drop in [pH]_cyt_ inhibits the activity of a soluble inorganic pyrophosphatase (de Graaf et al. 2006; Eaves et al. 2017). As these proteins are essential for many metabolic pathways in eukaryotic cells, this inhibition could have severe consequences for metabolism, which could contribute to triggering PCD.

Together, the available data point to cytosolic acidification in plant cells affecting numerous enzyme activities that could trigger entry into PCD. Thus, these relatively larger cytosolic acidification events may provide an additional layer of regulation over and above that used by normal physiological events such as regulation of growth. These larger-scale acidification events may be physiologically relevant in the context of some stresses, leading to PCD in eukaryotic cells.

## Several ways to achieve cytosolic acidification during PCD?

Having clear evidence that cytosolic acidification occurs during plant PCD, and that this drop in [pH]_cyt_ has serious consequences, prompts an important question: how is this acidification achieved? The timing of vacuolar breakdown in plant PCD varies greatly; in some PCD systems, it is sudden and early; in others, it is delayed and slow. Although the term vPCD (van Doorn et al. 2011) is frequently used in the literature, as mentioned earlier, there are at least 2 distinct patterns of breakdown of intracellular compartments: one, rapid (Fig. 1, E and F), and the other, delayed (Fig. 1, A to D, G to L).

In some systems, such as in root-cap cells undergoing dPCD and the *Papaver* SI system, which is an example of sPCD, cytosolic acidification precedes the breakdown of intracellular compartments (Fig. 1, G to J). In the *Papaver* SI-PCD system, [pH]_cyt_ dropped to pH 6.4 within 10 min, reaching pH 5.5 within 60 min after SI induction. Somewhat surprisingly, although vacuolar reorganization was observed within 15 min, vacuolar permeabilization and apparent breakdown were detected only 30 to 60 min after SI induction, which was well after the initial cytosolic acidification (Wilkins et al. 2015; Wang et al. 2020). A similar observation was reported during dPCD in Arabidopsis LRC cells; [pH]_cyt_ dramatically decreased ∼9 min prior to vacuolar collapse (Wang et al. 2024). Both these studies in very different PCD systems showed that cytoplasmic acidification occurs before tonoplast rupture. Thus, it appears that, contrary to expectation, vacuolar breakdown is not responsible for the early cytosolic acidification. This suggests that in some PCD systems, acidification may play an active role in driving PCD forward prior to the execution/cellular dismantling phase, especially as many enzyme activities will be altered by changes in [pH]_cyt_. Nevertheless, the later vacuolar breakdown and release of its contents into the cytosol will inevitably contribute to the later execution stages of PCD.

One of the earliest examples of measurement of large-scale acidification triggered during plant PCD, prior to vacuolar collapse and the execution/cellular dismantling phase, comes from the barley aleurone PCD system. Gibberellic acid triggers dramatic changes in the protein storage vacuoles (PSVs) of aleurone cells, which become acidic, lytic organelles. Unlike typical vacuoles, which are acidic, PSVs are unusual as they have a neutral lumen pH. However, within an hour of gibberellic acid addition, PSVs became acidic, and within ∼6 h, their pH dropped below 5.5 (Swanson and Jones 1996). Although this is not an example of cytosolic acidification, this early report indicates that H^+^ pumps are affected by upstream signals related to PCD.

As there have been few studies of cytosolic acidification being triggered early, before the execution stage of PCD, not much is known about how this is achieved. In the SI-PCD system, some of the events triggered by the receptor–ligand-type interaction have been elucidated using both *Papaver* and a transgenic “poppydopsis” system comprising *A. thaliana* expressing the *Papaver S*-determinants (Lin et al. 2015; Wang et al. 2020). The role of Ca^2+^, which has long been known to be a trigger for the SI response in *Papaver* (Franklin-Tong et al. 1997), has also been shown to act as an upstream trigger for PCD in this SI system (Thomas and Franklin-Tong 2004; Bosch and Franklin-Tong 2008). Moreover, as treatment with a calcium ionophore resulted in a decrease in [pH]_cyt_, this implicates Ca^2+^ as an upstream trigger for cytosolic acidification (Wilkins et al. 2015). Similar evidence of a close link between alterations in [Ca^2+^]_cyt_ and [H^+^]_cyt_ was found in the dPCD system of LRC in Arabidopsis roots (Wang et al. 2024; Fig. 1, G to J). In the *Papaver* SI-PCD system, another event identified as playing a pivotal role in the SI response includes an increase in ROS, accompanied by irreversible oxidation of many pollen proteins (Wilkins et al. 2011; Haque et al. 2020). Increases in ROS, which are recognized regulators of PCD in both plant and animal cells, are triggered by Ca^2+^ ionophores upstream of SI-induced PCD; the timing of these and other events involved in the SI-PCD system are outlined in several reviews (Wilkins et al. 2014; Wang et al. 2019, 2020, 2022b).

Evidence from the transgenic “poppydopsis” system offers insights into the mechanisms behind cytosolic acidification during SI. After SI induction, a rapid H^+^ influx at the PM was observed. A drop in pH_cyt_ occurred within 2 to 3 min, with distinct, mobile acidic patches as low as pH 5.6 appearing adjacent to the PM within a few minutes. These acidic patches became more pronounced and extensive, forming a peripheral acidic zone within 2.5 min, with clear evidence of a progression of acidification over time (Wang et al. 2022b). The use of membrane-impermeable Good's buffers provided evidence that SI induces an influx of protons and apoplastic pH, which is normally acidic, increased after SI induction. Together, these findings provide good evidence that early influx of extracellular H^+^ contributes to the SI-induced decrease in [pH]_cyt_ (Fig. 1, G and H).

Another clue comes from the finding that SI triggers rapid ATP depletion and that ATP depletion triggers cytosolic acidification in incompatible pollen tubes (Wang et al. 2022b). In the “poppydopsis” SI system, intracellular ATP dropped dramatically within 2 min of SI induction and had fallen further by 10 min, stabilizing at 24% of its original level. Artificially inducing ATP depletion in pollen tubes using 2-DG and antimycin A resulted in a rapid and large reduction in pH_cyt_, comparable with levels observed during SI within a similar timeframe (Wang et al. 2022b). Concurrently, distinctive acidic patches adjacent to the PM were observed, demonstrating that ATP depletion results in large, rapid decreases in pH_cyt_. Hence, changes in cellular energy levels can cause cytosolic acidification.

As mentioned earlier, control of transmembrane pH gradients involves complex interplay of H^+^ fluxes and energy metabolism (Wegner and Shabala 2020). As H^+^ efflux in pollen tubes is achieved by H^+^-ATPases, encoded by autoinhibited H^+^-ATPases (Fuglsang and Palmgren 2021), this suggests their inhibition in the SI response. H^+^ efflux was reduced and accompanied by [pH]_cyt_ acidification (due to failure to export H^+^) in Arabidopsis *aha6/8/9* mutants (Hoffmann et al. 2020). Moreover, acidosis would be predicted to further exacerbate the pumps’ energy supply as the free energy of ATP decreases with declining pH (Davies et al. 1993). As it is well established that decreases in ATP synthesis are usually caused by mitochondrial defects, this implicates the mitochondria as an early target of signals for PCD. In the *Papaver* SI response, increases in ROS and oxidation of many pollen proteins, including a predicted ATP synthase subunit (Haque et al. 2020), are observed in incompatible pollen, as well as rapid release of cytochrome *c* (Thomas and Franklin-Tong 2004) and dramatic changes in mitochondrial morphology (Geitmann et al. 2004), suggesting early targeting of mitochondria during SI, which is likely to impact ATP synthesis. In the LRC system, transient increases in [Ca^2+^]_cyt_ and [H^+^]_cyt_ concentrations, triggered by using the drug CCCP, were sufficient to trigger PCD in differentiated root-cap cells (Wang et al. 2024). This drug is an oxidative phosphorylation inhibitor and a mitochondrial uncoupler that increases membrane permeability to protons, leading to a disruption in the mitochondrial membrane potential (Δ*ψ*_m_). This suggests mitochondrial involvement in the LRC PCD system.

**Figure 2.**
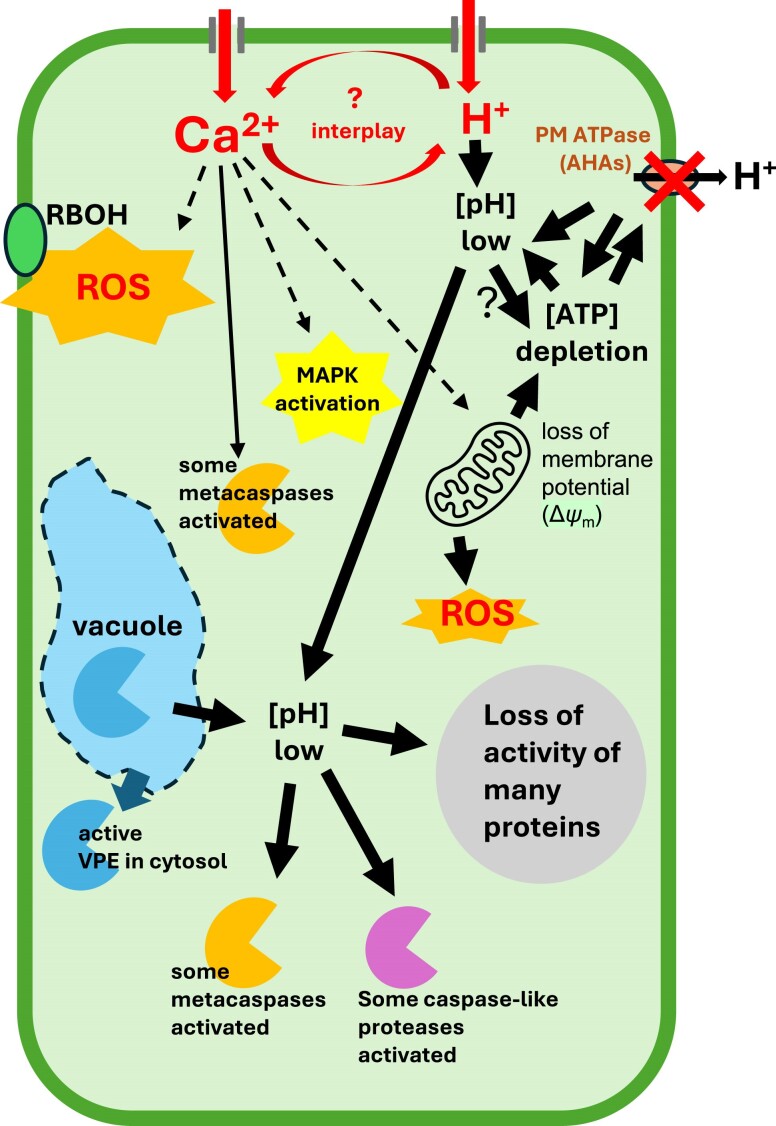
Potential targets (and interplay between them) of Ca^2+^ and H^+^ influx leading to PCD. Both the very early stages of PCD, involving Ca^2+^ and H^+^ alterations (top), and the later stages involving vacuolar breakdown (bottom), are indicated. See text for details.

The loss of mitochondrial integrity, along with the consequential loss of Δ*ψ*_m_ and reduction in ATP synthesis, has been suggested to be a critical step in some plant PCD systems (Curtis and Wolpert 2004). Moreover, ATP depletion can affect pH_cyt_ in animal cells, as depletion of metabolic energy substrates inhibits the antiporter activity due to a modulation of an intracellular proton-dependent regulatory mechanism (Cassel et al. 1986). Although the role of the mitochondria in plant PCD is controversial, the data point to early mitochondrial dysfunction playing an important role in some types of plant PCDs, with their role in the production of both ROS and ATP impacting cellular functions (Vianello et al. 2007; Møller et al. 2021). Mitochondrial defects are often observed during plant PCD. In vPCD, they occur late, after vacuolar rupture (van Doorn et al. 2011). In other systems, they occur early, as seen in both the root and pollen PCD systems and in the HR. It is thought that mitochondria are likely involved in some forms of plant PCD, particularly where early rupture of the vacuole is not triggered (Vianello et al. 2007). While reports of ATP depletion in plants are less common, it has been observed during various forms of PCD (Tiwari et al. 2002; Krause and Durner 2004; Hatsugai et al. 2012; Wang et al. 2022b), suggesting that a reduction in cellular ATP levels may be an early signal to trigger PCD in plants. A number of studies suggest that mitochondria may play a key role in integrating PCD signals, with its redox status potentially regulating a critical balance between life and death (Mur et al. 2007). Mitochondrial dysfunction and general cellular metabolic dysfunction are likely to lead to PCD if unregulated. We will not discuss mitochondrial dysfunction further here, as it is a topic in its own right; the reader is directed to Mur et al. (2007) and Wang et al. (2022a) who review mitochondrial responses during HR-PCD. All these events are likely to be intertwined (Fig. 2); establishing how [Ca^2+^]_cyt_ and [H^+^]_cyt_ signal to achieve these cellular alterations and how these changes mechanistically contribute to driving plant PCD forward will be an important challenge for the future.

## A model for the interplay between Ca^2+^ and H^+^ influx leading to PCD

The intracellular pH of plant cells is strictly controlled. Figure 2 explores how some of the cellular components might interact to regulate PCD in systems where early cytosolic acidification has been observed. As not many studies have reported acidification, this model is necessarily speculative and is based largely on what is known about changes in these dynamics in non-PCD systems. Ca^2+^ is well established to regulate many types of PCD. Cytosolic acidification is likely to be caused by failure to export H^+^. The activity of PM H^+^-ATPases, which regulate H^+^ efflux, is critical for the homeostasis of intracellular [pH]_cyt_. Inhibition of this H^+^ pump activity will cause acidosis. There is evidence for a fundamental link between Ca^2+^ and H^+^ dynamics in plant cells, with interplay between the 2, although how this is achieved is largely unknown. The control of transmembrane pH gradients involves interplay between H^+^ fluxes and energy metabolism. ATP depletion can result in large decreases in pH_cyt_. Because the free energy of ATP declines with declining pH, cytosolic acidification will worsen the H^+^ pumps’ energy supply. Mitochondria are implicated, as decreases in ATP synthesis are usually caused by mitochondrial defects. The loss of mitochondrial membrane potential and consequent ATP depletion is likely to have important consequences for cellular survival. Increases in intracellular Ca^2+^ will have consequences for the activity of many enzymes, including RBOHs, MAPKs, and Ca^2+^-dependent metacaspases. Decreases in cytosolic pH will also have consequences for the activity of many enzymes, including MAPKs, pH-sensitive metacaspases, and caspase-like proteases that require an acidic pH for activity. Together, these events could drive the cell into PCD. Later events, involving cellular dismantling, including loss of vacuolar integrity with release of its acidic contents including vacuolar proteases into the cytosol, are likely to contribute to later execution steps of PCD. This could further reinforce or stabilize the intracellular acidification leading to the execution stages of PCD.

## Breakdown of intracellular compartments

During PCD, in addition to the loss of vacuolar and mitochondrial integrity, other organelles become permeabilized. Although this is not the focus of our review, we mention this for completeness. Alterations to intracellular organelles have been observed in a number of plant PCD systems, forming part of the cellular dismantling phase. The breakdown of organelles appears to be quite variable, occurring at different rates, depending on the specific system and context. There appear to be several distinct modes of intracellular breakdown, and various attempts have been made to describe their progression in different plant PCD systems; see, for example, Lam (2004). Apoptosis-like cell death in plants is rapid, beginning with the degradation of the nucleus and subsequent incomplete breakdown of cellular organelles (Fukuda 2000). Another rapid type of plant PCD, vPCD, is characterized by the early disruption of the central vacuole, leading to the sudden release of lytic enzymes into the cytoplasm (Fukuda 2000). In other systems, cellular dismantling is delayed, and metacaspase-dependent autophagy is used to execute some forms of this type of PCD. Autophagy is thought to play a role during some types of vPCD, as it provides a mechanism to sequester and deliver cellular components to the vacuole for degradation, thereby enabling controlled cellular self-disassembly. A detailed, systematic analysis of cell death in the apical meristem of primary roots of Arabidopsis during water stress-induced PCD revealed key features of autophagic cell death, including an increase in vacuole size, degradation of organelles, and collapse of the tonoplast and the PM (Duan et al. 2010). Autophagy may also act to maintain the energy status of dying cells for some time, allowing cells to accomplish properly orchestrated and controlled PCD (Minina et al. 2013, 2014). Recently, a temporal analysis of several decompartmentalization events during dPCD execution in the root cap was described. Briefly, this analysis showed the rapid disintegration of mitochondria, followed soon after by permeabilization of the ER and nuclear envelope, with vacuolar breakdown occurring later, accompanied by loss of PM integrity (Wang et al. 2024). In other systems, for example, in senescence-induced PCD, breakdown is very slow, with chloroplasts degraded initially, followed by the disruption of the nucleus and vacuole at the end of cell death (Smart 1994; Fukuda 2000). It seems likely that the regulation and temporal pattern of these later events are more variable and designed to suit the needs of the specific PCD system and its purpose.

## Ca^2+^ and pH play pivotal roles in different PCD systems

In summary, although there are many differences between various plant PCD systems, they appear to fall into 2 main categories. One involves a rapid “destructive” vacuolar collapse that causes (assumed) rapid cytosolic acidification. This results in the release of VPE (a YVADase/caspase-1-like protease) that is active at low pH into the cytosol (Fig. 1, E and F). Only when this happens (accompanied by assumed cytosolic acidification that would allow VPE activity) would this type of PCD start to enter the execution phase. The other category, where vacuole breakdown is delayed, is often triggered by Ca^2+^ influx via diverse Ca^2+^ channels and/or an early cytosolic acidification, although in many cases, the latter has not been measured (Fig. 1, A to D, G to L). There are several scenarios, as some metacaspases normally present in the cytosol, with pH optima of pH ∼7 (e.g. AtMC4), are inactive at low physiological [Ca^2+^]_cyt_ and would be activated by high [Ca^2+^]_cyt_ caused by Ca^2+^ influx (Fig. 1, A to D, K, and L). Other metacaspases/caspase-like enzymes (e.g. AtMC9) are present in the cytosol but are inactive at physiological pH ∼7 and require an acidic pH to allow activation. Two possibilities exist here: acidification via H^+^ influx or acidification by vacuolar breakdown; this should be examined. Some plant PCD systems utilize different caspase-like enzymes, e.g. a yet to be identified enzyme with DEVDase activity in the *Papaver* SI system and a cathepsin B (AtCathB3) involved in abiotic stress-induced PCD in Arabidopsis, which also has DEVDase activity (Ge et al. 2016). These are present in the cytosol, but as they have optimal activity at acidic pH (pH 5 to 5.5), they will remain inactive until the pH becomes acidic. In the LRC system, it is not known which proteases are involved, but the pattern of acidification and later vacuolar breakdown appears similar to the *Papaver* SI system and these 2 PCD systems involve both Ca^2+^ influx and early cytosolic acidification (Fig. 1, G to J). It will be interesting to ascertain if the pattern-triggered or effector-triggered immune responses also involve early cytosolic acidification prior to vacuolar breakdown. Thus, in several systems, the evidence points to Ca^2+^ and H^+^ alterations playing pivotal roles in regulating PCD.

## Questions to be addressed in the future

There is now good evidence that Ca^2+^ influx and cytosolic acidification are widely used to regulate PCD in plants. It is evident that an initial [pH]_cyt_ drop is not universally achieved by vacuolar breakdown, and this phenomenon may be more widespread if investigated further. A key question to be ascertained in future studies is how, exactly, the dramatic drop in cytosolic pH is achieved, and how it is regulated. As mentioned earlier, it is well established that the intracellular pH of living cells is strictly controlled at the PM and by each compartment. Although appreciation of major changes in cytosolic pH is relatively recent in the PCD field, there is a wealth of literature on pH regulation during normal growth and development involving channel activity. A focus on ion transport during PCD may shed some light on this. It has been observed that an early response in anoxic cells is a rapid, small (∼0.5 pH units) cytoplasmic acidification and that the intracellular pH can then drop further due to an energy shortage, in concert with a general breakdown of transmembrane gradients (Felle 2005). Thus, cytosolic acidification will eventually result in cell death unless the cell can utilize an alternative energy source.

It is important to take on board the idea that pH changes may not be uniform across the cell and its various intracellular compartments (apoplast, cytosol, or vacuole). Now that suitable tools are available, imaging cytosolic pH using pH-sensitive probes will hopefully provide insights into alterations during PCD in different systems. There has been a lack of studies assessing the role of H^+^ transporters such as PM H^+^-ATPases and other channel activities that can alter [pH]_cyt_, as potential regulators of PCD. This will be an important area for future research. Other areas that would be valuable to investigate include crosstalk between pH and Ca^2+^ as well as ROS during PCD.

We have not provided an extensive review of all the features of PCD, nor do we cover many of the phenomena associated with the triggering and execution of PCD here in depth. This includes crosstalk between organelles (Van Aken and Van Breusegem 2015), breakdown of organelles, and identification of proteases released by these processes and how they are involved in PCD (Thomas and van der Hoorn 2018; van der Hoorn and Rivas 2018; Buono et al. 2019). Although much progress has been made in recent years, some of the areas relating to PCD remain quite a mystery, and much remains to be elucidated.

## Concluding remarks

Although a wealth of data has been gathered over the last couple of decades, there are still gaps in our knowledge of how PCD is achieved at a mechanistic level. Comparing what we know about the execution of PCD in different systems is challenging. However, although the triggers and responses may be different, it appears that many of the downstream signals and intracellular mechanisms involved in both sPCD and dPCD are similar. Thus, there does not seem to be a clear distinction between the mechanisms utilized by these 2 types of PCDs. Taken together, and despite differences between the temporal events and gaps in our knowledge about how some of these processes are mechanistically achieved, the current knowledge suggests that there are some key events that are common to many PCD systems.

Over the last few decades, investigation of the cell biology of processes involved in PCD in various plant systems has revealed several key hallmark features, especially in the signaling networks involved. Ca^2+^ as a second messenger has been long established, and ROS is another critical player. An interesting recent review has compared different types of cell deaths in animal cells (apoptosis, necroptosis, pyroptosis, and ferroptosis) with the HR in plant cells, drawing attention to key regulators, formation of “deathosome” structures, and impact on membrane integrity (Maekawa et al. 2023). They point out that alteration of endomembrane integrity represents a crucial step in immunogenic cell death signaling in both animals and plants. Here, we have attempted to draw several strands of evidence relating to an often overlooked or assumed aspect of PCD, namely, cytosolic acidification. We hope that highlighting this topic will stimulate further research in the plant PCD field to ascertain if acidification is triggered in other plant PCD systems and how this is achieved. This raises the question of whether there are further similarities and whether there are common mechanisms and/or fundamental evolutionary links underlying them, either having evolved from a common ancestral point or by recruitment of key mechanisms.

Despite major progress in many areas, our understanding of the execution of PCD in plants remains fragmentary. This is complicated by the fact that there seem to be several forms of PCD based on morphological observations (see, for example, van Doorn et al. 2011). However, a review of similarities (and differences) between cell biological and biochemical PCD responses in plants has, to date, not been properly attempted (Kacprzyk et al. 2024). Despite the huge difficulties in comparing the many different PCD systems, we believe that attempting to find further commonalities between various plant PCD systems and understanding the signaling networks regulating them will strengthen our knowledge of how these mechanisms operate and how they evolved.

## Data Availability

No new data were generated in support of this study.

## Dive Curated Terms

The following phenotypic, genotypic, and functional terms are of significance to the work described in this paper:

gibberellic acid CHEBI: CHEBI:86644

PTI Gramene: pattern-triggered immunity
PTI Araport: pattern-triggered immunity
